# *Cis*-Effects Condition the Induction of a Major Unfolded Protein Response Factor, *ZmbZIP60*, in Response to Heat Stress in Maize

**DOI:** 10.3389/fpls.2018.00833

**Published:** 2018-06-29

**Authors:** Zhaoxia Li, Renu Srivastava, Jie Tang, Zihao Zheng, Stephen H. Howell

**Affiliations:** ^1^Plant Sciences Institute, Iowa State University, Ames, IA, United States; ^2^Department of Genetics, Development, and Cell Biology, Iowa State University, Ames, IA, United States; ^3^Department of Agronomy, Iowa State University, Ames, IA, United States

**Keywords:** maize, unfolded protein response, *ZmbZIP60*, transposable element, heat stress

## Abstract

Adverse environmental conditions such as heat and salt stress create endoplasmic reticulum (ER) stress in maize and set off the unfolded protein response (UPR). A key feature of the UPR is the upregulation of *ZmbZIP60* and the splicing of its messenger RNA. We conducted an association analysis of a recombinant inbred line (RIL) derived from a cross of a tropical founder line, CML52 with a standard temperate line, B73. We found a major QTL conditioning heat-induced *ZmbZIP60* expression located *cis* to the gene. Based on the premise that the QTL might be associated with the *ZmbZIP60* promoter, we evaluated various maize inbred lines for their ability to upregulate the expression of *ZmbZIP60* in response to heat stress. In general, tropical lines with promoter regions similar to CML52 were more robust in upregulating *ZmbZIP60* in response to heat stress. This finding was confirmed by comparing the strength of the B73 and CML52 *ZmbZIP60* promoters in transient maize protoplast assays. We concluded that the upstream region of *ZmbZIP60* is important in conditioning the response to heat stress and was under selection in maize when adapted to different environments.

**Summary:** Heat stress has large negative effects on maize grain yield. Heat stress creates ER stress in maize and sets off the UPR. We searched for factors conditioning heat induction of the UPR in maize seedlings by conducting an association analysis based on the upregulation of unspliced and spliced forms of *ZmbZIP60* mRNA (ZmbZIP60u and ZmbZIP60s, respectively). ZmbZIP60u was upregulated more robustly by heat stress in the tropical maize line, CML52, than in B73, and a major QTL derived from the analysis of RILs from a cross of these two lines mapped in the vicinity of *ZmbZIP60*. We conducted a *cis/trans* test to determine whether the QTL was acting as a *cis* regulatory element or in *trans*, as might be expected for a transcription factor. We found that the QTL was acting *in cis*, likely involving the *ZmbZIP60* promoter. *ZmbZIP60* promoters in other temperate and tropical lines similar to CML52 showed enhanced expression of ZmbZIP60u by heat. The contribution of the CML52 promoter to heat induction of *ZmbZIP60* was confirmed by analyzing the CML52 and B73 promoters linked to a luciferase reporter and assayed in heat-treated maize protoplasts.

## Introduction

Maize is the most widely produced crop in the world. It has been adapted to many different environments and now faces changing climate conditions. Adverse environmental conditions present a major constraint in preventing crops such as maize from reaching their genetic potential. It has been estimated that each year 15–20% of the potential maize production is lost due to drought and heat (Lobell et al., 2011; Makarevitch et al., 2015) and that each degree increase in global mean temperature reduces maize yields worldwide by 7.4% (Wang et al., 2017).

Through domestication, many agronomic, plant architecture, and seed quality traits in maize have been subject to selection at thousands of loci (Tian et al., 2009). In the process, small regions surrounding selected genes have been substantially reduced in genetic diversity (Meyer and Purugganan, 2013). The reduction in genetic diversity in the regulatory elements limits the adaptability of maize to different environmental conditions (Freeman and Herron, 1998). Both *cis-* and *trans-*regulatory element variation contribute to this diversity, however, *cis-*regulatory variation is more common for both steady-state and stress-responsive expression differences (Waters et al., 2017).

Limited adaptability can exacerbate the effects of the environment on fragile cellular processes such as the folding of proteins in the endoplasmic reticulum (ER) creating a condition called ER stress (Thomashow, 1999; Bray, 2004; Liu et al., 2007; Deng et al., 2011). This happens when the demand for protein folding under adverse environmental conditions exceeds the cell’s capacity, setting off the unfolded protein response (UPR). The UPR is a homeostatic response to lighten the load of misfolded proteins in the ER and to protect plants from further stress (Deng et al., 2013a; Howell, 2013). The UPR communicates the status of protein folding in the ER to the nucleus. In Arabidopsis, the UPR signaling pathway has two arms: one arm involves ER membrane-associated transcription factors (AtbZIP17 and AtbZIP28) that are mobilized to the nucleus in response to stress and another arm that involves a dual protein kinase, RNA-splicing factor, IRE1, and its target RNA, AtbZIP60 (Deng et al., 2013a; Howell, 2013; Korner et al., 2015).

Both arms of the UPR are involved in abiotic stress responses where they have been examined most extensively in Arabidopsis. Loss-of-function mutations in AtbZIP17 or in factors involved in its processing, such as S1p (Site-1 protease), are salt sensitive, while overexpression of AtbZIP60 confers more tolerance to salt stress (Liu et al., 2007; Deng et al., 2013b; Henriquez-Valencia et al., 2015). Double *ire1a ire1b* mutations in Arabidopsis are more sensitive to ER stress agents such as dithiothreitol (DTT) and pollen production in the double mutant is heat sensitive (Deng et al., 2016).

The UPR in maize seedlings is induced by heat stress, i.e., by exposing seedlings to elevated temperatures (Li et al., 2012). There are many studies on heat stress in maize that differ in a number of ways including whether heat stress is applied as heat shock or simply as growth at elevated temperature (Ristic et al., 1998; Qu et al., 2017). Reproductive development in maize is particularly vulnerable to heat stress. Elevated temperature during pollination has profound effects on maize pollen shed and viability (Schoper et al., 1987; Lizaso et al., 2018) and at later stages on grain filling (Badu-Apraku et al., 1983; Wilhelm et al., 1999). Heat stress also affects photosynthesis during vegetative growth (Berry and Bjorkman, 1980; Edwards et al., 2001; Crafts-Brandner and Salvucci, 2002; Sinsawat et al., 2004). Photosynthesis is sensitive to heat stress largely attributed to the inactivation of Rubisco and denaturation of Rubisco activase at elevated temperature (Law and Crafts-Brandner, 1999; Salvucci et al., 2001; Crafts-Brandner and Salvucci, 2002; Salvucci and Crafts-Brandner, 2004) For reasons which have not been further explored, heat tolerance varies among inbred lines (Chen et al., 2012; Cairns et al., 2013; Naveed et al., 2016), particularly in the comparison between temperate and tropical lines (Edreira and Otegui, 2012).

In this paper, we examined the induction of the UPR by heat stress in maize seedlings. We evaluated the ability of several maize inbred lines belonging to different sub-families to upregulate and splice *ZmbZIP60* mRNA in response to heat stress. We found that the upstream region of *ZmbZIP60* plays an important role in regulating the gene’s response to heat stress and is under selection for maize adapted to different environments.

## Results

### QTL Analysis of Variation in the UPR

The upregulation of the canonical ER stress response genes has served as a molecular signature for the UPR. We examined two other outputs of the UPR, the production of unspliced and spliced forms of *ZmbZIP60* mRNA [ZmbZIP60u and ZmbZIP60s (spliced form, mobilized to the nucleus), respectively] that are upregulated by ER stress agents, such as tunicamycin and dithiothreitol, and also by heat (Li et al., 2012). We observed greater heat induction of ZmbZIP60u in four tropical inbreds, Ki3, CML69, CML103, and CML322 compared to the temperate lines, B73 and IL14H, (**Supplementary Figure S1**). We also observed higher levels of ZmbZIP60s in response to heat stress in the tropical lines. Therefore, we utilized ZmbZIP60u and ZmbZIP60s as biomarkers to study the differences in UPR in tropical vs. temperate lines.

**FIGURE 1:**
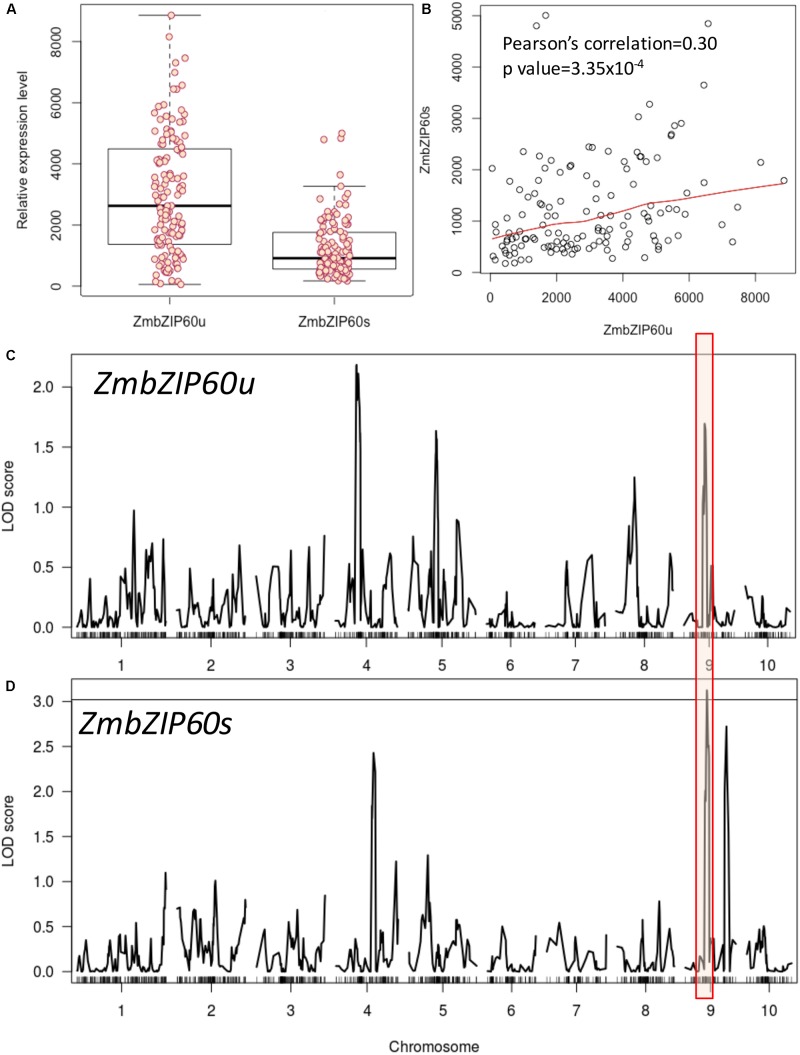
QTL analysis of heat-induced ZmbZIP60 expression in NAM RILs. RILs were derived from a cross of B73 × CML52, and relative gene expression values were determined by ImageJ measurements of band density from a RT-PCR analysis. **(A)** Box plot of the relative heat-induced gene expression levels in the various RILs for unspliced form of ZmbZIP60 mRNA (ZmbZIP60u) and the spliced form (ZmbZIP60s). Dark horizontal lines are the means of the gene expression levels, the box indicates the standard deviation values and the whiskers are the limits of variation in the levels of expression. **(B)** Correlation between the heat-induced levels of ZmbZIP60u and ZmbZIP60s in the various RILs. **(C)** Association analysis to map heat-induced ZmbZIP60u QTLs onto the maize B73 genome. Major peak boxed in red marks the vicinity of the ZmbZIP60 gene. **(D)** Similar association analysis mapping the heat-induced ZmbZIP60s expression QTLs. Line indicates significant LOD 3 score.

To search for factors conditioning *ZmbZIP60* induction in response to heat stress, we analyzed a set of RILs from the nested association mapping (NAM) population derived from a cross of B73, a temperate line, x CML52, a lowland tropical yellow maize inbred (McMullen et al., 2009). The heat induction of ZmbZIP60u varied more widely among the NAM RILs than the induction of ZmbZIP60s (**Figure 1A**). The levels of ZmbZIP60u appeared to contribute in part to the levels of ZmbZIP60s as demonstrated by the correlation between the induced expression of ZmbZIP60u and ZmbZIP60s (correlation coefficient of 0.30) (**Figure 1B**).

An association analysis was conducted to map heat-induced *ZmbZIP60* QTLs onto the maize B73 genome. Heat induction of ZmbZIP60u showed broad heritability estimated to be 0.79 while the heritability of heat induction of ZmbZIP60s was estimated to be 0.83. The association analysis showed that 46 SNPs contributed significantly to the upregulation of ZmbZIP60u while 48 SNPs contributed to ZmbZIP60s (**Figures 1C,D**). Phenotypic variation explained by individual SNPs ranged from 4 to 10% for ZmbZIP60u and from 3.0 to 17% for ZmbZIP60s. Interestingly, there was a significant association haplotype block located on chromosome 9, in the vicinity of *ZmbZIP60*, suggesting that polymorphisms likely upstream of *ZmbZIP60* in the tropical line contribute significantly to the upregulation of *ZmbZIP60* expression in response to heat stress.

### *Cis/Trans* Test

**FIGURE 2:**
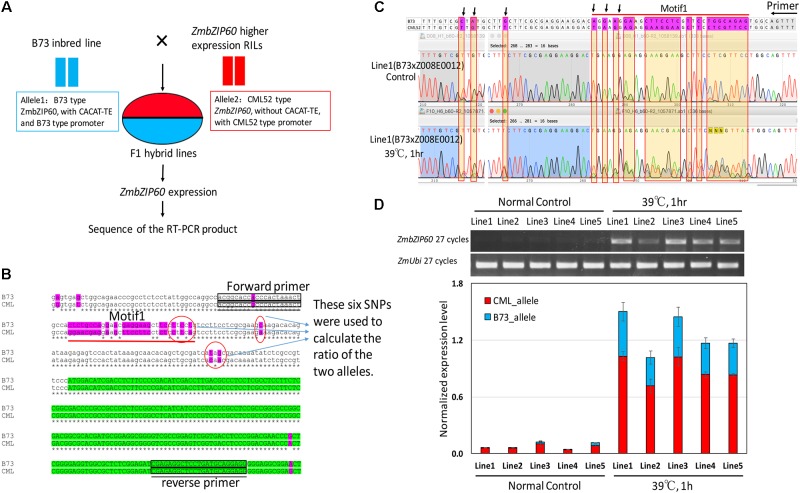
*Cis/trans* test of heat-induced *ZmbZIP60* expression. **(A)** F1 hybrid lines were obtained from crosses of B73 × high expressing RILs with CML52 type promoters. *ZmbZIP60* expression was heat induced and the RT-PCR product was sequenced to determine the relative expression of the two alleles. **(B)** DNA sequence analysis of the B73 type and the CML52 type alleles. Six SNPs in the 5′-UTR were used to distinguish the contribution of the two different alleles in the RNA transcript population. Green highlights the coding region and purple highlights sequence variation between the alleles. Boxes indicate the location of the primers used in the RT-PCR analysis. **(C)** Sequence profile of RT-PCR products from the analysis of hybrid line 1 derived from a cross of B73 × Z008E0012. RNA extracted from seedlings incubated at 26°C served as a control, while the experimental sample was extracted from seedlings incubated for 1 h at 39°C. The area under the peaks for the SNPs (pointed out by arrows) was measured and the ratio of the two different alleles was calculated. **(D)** RT-PCR analysis of the RNA extracted from control hybrid seedlings incubated at 26°C and those subjected to heat treatment (39°C, 1 h). Bar graph represents the normalized contribution of the two alleles (CML type and B73 type) to the RNA population in the control and heat-treated seedlings.

To determine whether the QTL in the vicinity of *ZmbZIP60* functions *in cis* or *in trans* with respect to *ZmbZIP60* induction, a *cis/trans* test was performed. To conduct the test, F1 hybrids were selected from crosses of B73 to five different RILs (Z008E0012, Z008E0105, Z008E0127, Z008E0135, and Z008E0143), which bore *ZmbZIP60* from CML52 and that induce ZmbZIP60u to high levels in response to heat. In the F1 lines, one *ZmbZIP60* allele was derived from B73 and the other from CML52 (**Figure 2A**). The test was to determine whether induction conditions act *in trans* to elevate the expression of both alleles equally or whether the response acts *in cis* to raise the expression of one allele over the other. To carry out the test, six SNPs in the 5′-UTR of *ZmbZIP60* were used to distinguish the RNA transcripts from the two different alleles (**Figure 2B**). The ratios (CML52/B73 allele) of the six SNPs were used to calculate the relative abundance of the transcripts derived from the two different alleles. Before heat stress, ZmbZIP60u expression was quite low, but after heat stress, ZmbZIP60u expression increased 9.1∼22-fold in the various lines. Both alleles were induced, however, the contribution was unbalanced. CML52 allele contributed more to the heat-induced transcript population, contributing nearly 70% of the total transcripts (**Figures 2C,D**). We interpret this to mean that the elevated heat induction response of *ZmbZIP60* is mostly due to *cis* elements that regulate expression of the gene.

### The Analysis of the Upstream Region of *ZmbZIP60*

**FIGURE 3:**
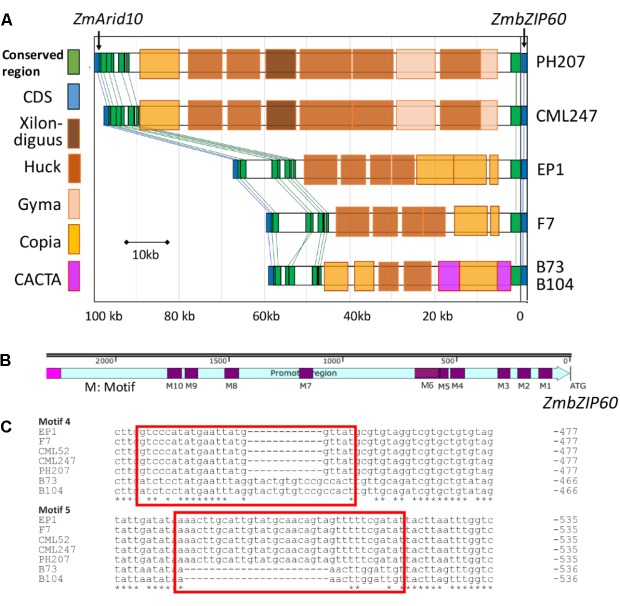
Diversity in the upstream region of *ZmbZIP60.* **(A)** Transposable element (TE) content in the far upstream region of *ZmbZIP60* in six different maize inbred lines. Upstream region spans the region from the start of transcription (*ZmbZIP60*) to the upstream *ZmArid10* gene. Distances are indicated in base pairs. TEs are color coded according to the legend. **(B)** Near upstream region (2200 bp) of *ZmbZIP60* in the six different maize inbred lines. Indicated are ten different sequence motifs (marked M1-10) representing indels in the promoter region. **(C)** Sequence motifs 4 and 5 were used to distinguish B73 type promoters from CML type (as in CML247) promoters.

The contribution of *cis* effects to the heat induction of *ZmbZIP60* gave us cause to look upstream from the gene for possible control elements. The upstream sequences for *ZmbZIP60* in six reference maize lines (B73, B104, EP1, F7, CML247, and PH207) were quite different in comparing tropical and temperate lines (**Figure 3A**). In the B73 and B104 lines, the distance between *ZmbZIP60* and the upstream gene *ZmArid10* was about 56kb and was 58 and 66 kb for two European founder lines F7 and EP1, respectively. However, the distance of ZmbZIP60 to the upstream gene, *ZmArid10*, was much longer in the tropical line CML247 (96 kb) and in PH207, belonging to the iodent germplasm (98 kb). The differences can be attributed to the various transposable elements (TEs) present in this region. For example, in CML247 and PH207, there are two gyma TEs, five huck TEs, one xilon-diguus TE, and one copia TE in the upstream region, all of which are Class I retroTEs. However, in the same region in B73 and B104 there are CACTA, a Class II TE, and copia TEs. A 12-bp sequence (-CTTTGCCGAGTG-) was also found repeated 10 times from −2304 to −2588 bp upstream in B73, followed by the CACTA TE which was disrupted by a Class I Copia TE. In EP1 and F7, a Copia TE was inserted −5842 bp upstream from the start of translation and a gyma element was inserted in −6235 bp of CML247 and PH207, respectively.

**FIGURE 4:**
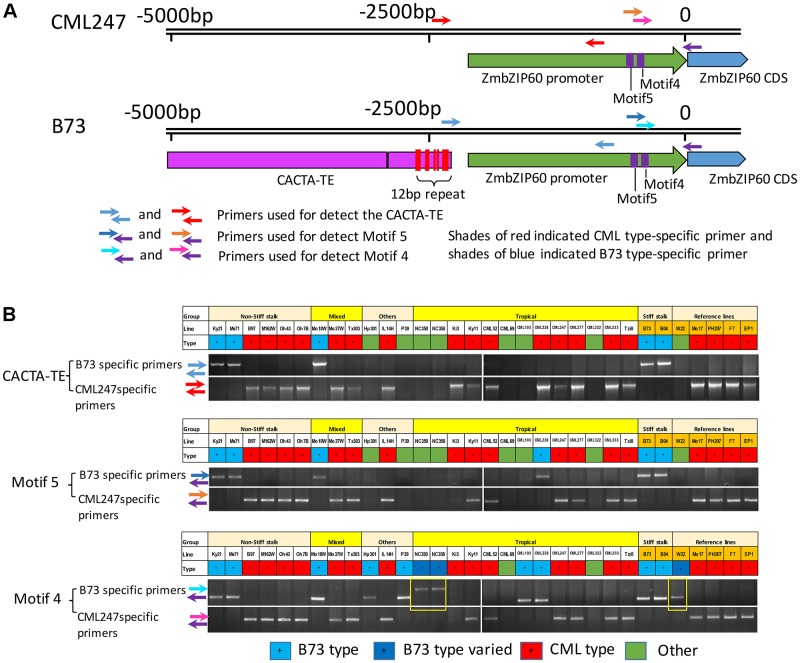
Genotyping and classifying *ZmbZIP60* promoters. **(A)** 5000 bp upstream region of *ZmbZIP60* showing location of primers for genotyping and classifying promoters as being B73 or CML type. Note CACTA-TE and 12 bp repeats in the B73 upstream region. **(B)** Genotyping the NAM founder and various inbred maize lines. Primer set 1 was used to determine the presence of the CACTA TE, primer set 2 was employed to identify motif 4 and primer set 3 was used for motif 5. Based on the presence or absence of these elements, all 32 maize lines could be categorized into one of three groups, B73 type (blue), CML247 type (red), and other type (green) The boxes outlined in yellow show B73 type motif 4 in lines NC350, NC358, and W22, however, they are located differently from the start of transcription than in B73. The designation “Other” indicates the promoter could not be classified by this criterion.

The near upstream region of *ZmbZIP60* (∼1.8 kb) in the reference lines and CML52 was marked by a number of indels. Ten major indels in this region were found in the comparison between B73/B104 and the other four lines, and they were named motif 1–10 according to their distance from the start of translation (**Figures 3B,C** and **Supplementary Figure S2**). The *ZmbZIP60* promoters in 25 different NAM founder lines (Liu et al., 2003; McMullen et al., 2009), were characterized in the same way using three sets of primers, one specific to B73 and another specific to CML247 (**Figure 4A**). Based on the presence or absence of the CACTA TE and motifs 4 and 5, all 32 maize lines could be categorized into one of three groups, B73 type, CML247 type and other type (**Figure 4B**). Maize germplasm can be categorized as belonging to Iowa Stiff Stalk Synthetic (BSSS) and Lancaster, represented by B73 and Mo17 (Mumm and Dudley, 1994), or to one of four groups: non-Stiff Stalk, Tropical or Semitropical, Stiff Stalk, and a mixed group (Liu et al., 2003). In this study, inbred lines represent non-Stiff Stalk, Tropical or Semitropical, Stiff Stalk, and a mixed group background. According to the presence of CACTA-TE and motif 5, five lines (Ky21, Ms71, Mo18w, B104, and B73) were classified as B73 type, while 18 maize lines belonged to CML type, without the CACTA-TE and CML247 motif 5 in their promoters. They included five non-stiff stock lines (B97, M162w, Oh43, Oh7B, and Mo17), two mixed lines (Mo37w and Tx303), one other line (IL14H), eight tropical lines (Ki3, Ki11, CML52, CML247, CML277, CML333 and Tzi8, iodent germplasm PH207), and two European founder lines (F7 and EP1). The presence of the CACTA-TE was linked with motif 5, except in one line, CML228. This line lacked the CACTA-TE but had a B73 motif 5 in its promoter. Eight lines were classified as other (green) based on these two sets of primers. Using primer set 3 (for motif 4), nine lines could be classified as B73 type (with B73 type motif 4), 18 lines as CML type (with CML247 type motif 4), three lines (NC350, NC358 and W22) had the B73 type motif 4, but the distance from ATG to motif 4 was somewhat different than B73, nonetheless, they were classified as B73-like. Only CML69 and CML322 could not be classified by these criteria.

### Association of Heat Stress Response With *ZmbZIP60* Promoter Features

**FIGURE 5:**
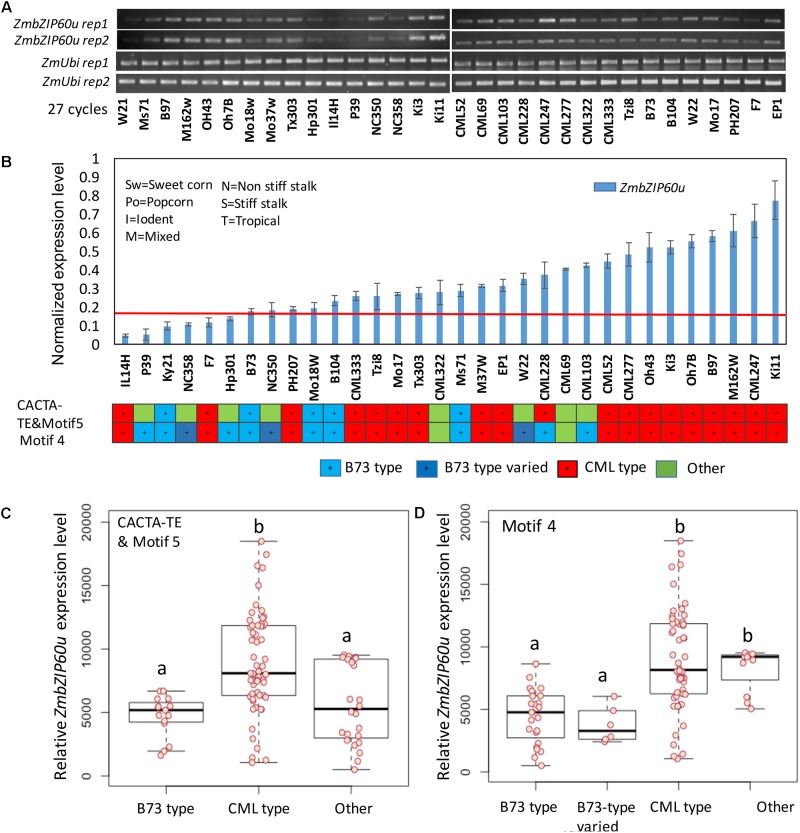
Correlation between promoter type and the level of heat-induced ZmbZIP60u expression. **(A)** RT-PCR analysis of the expression of ZmbZIP60u expression in 32 various maize NAM founder and reference lines. **(B)** The levels of expression were evaluated using ImageJ, and normalized with respect to the levels of ubiquitin. The maize lines were rank ordered by their level of heat-induced ZmbZIP60u expression. Also indicated are the heterotic groups in which these lines are classified. Error bars = SD for four biological replicates including the two shown above. **(C)** Box plot showing the heat-induced levels of ZmbZIP60u expression in lines characterized as having B73, CML, or other type promoters based on CACTA-TE and motif 5. Horizontal black line is the mean level of expression, the box indicates the standard deviation and the whiskers show the maximum variance. **(D)** As in **C**, the box plot shows the heat-induced levels of ZmbZIP60u expression in lines with type promoters based on motif 4. One-way ANOVA analysis and multiple comparisons were performed using Tukey test of the data in **C,D** and listed in **Supplementary Figure S3**. Letters a and b indicate significant differences between groups.

Following heat treatment of seedlings at 37° C for 1 h, significant differences in ZmbZIP60u upregulation were observed between the B73 and CML promoter types. Among the 19 lines that belong to CML type, based on CACTA-TE and motif 5 specific primers, 16 lines had higher expression levels (**Figure 5**). Among the B73 type (blue), all five lines had lower expression levels. Using motif 4 as a guide, among the lowest expressing 13 lines, 9 were B73 type (blue). The correlation between *ZmbZIP60* induction levels and the presence of CACTA-TE/motif 5, motif 4 and the interaction of motif 4 with the CACTA-TE/motif 5 was evaluated using a linear model. In general, the classification of the promoter region was highly correlated with the induced expression levels of ZmbZIP60u (**Figure 5**). The presence of motif 4 in the promoter was more highly correlated with induced expression than motif 5, but that may be partially due to differences in the population size for the different motifs (including three additional B73-like lines NC350, NC358, and W22). Nine lines with motif 4 had the lower expression levels than other lines, especially when compared to CML type, i.e., the lines with the same motif as CML247 (**Figure 5**). The presence of CACTA-TE, motif 4 and 5 and their interaction was highly correlated with the enhanced expression levels of *ZmbZIP60* after heat treatment (**Supplementary Figure S3**).

### Promoter Activity in Maize Protoplasts

**FIGURE 6:**
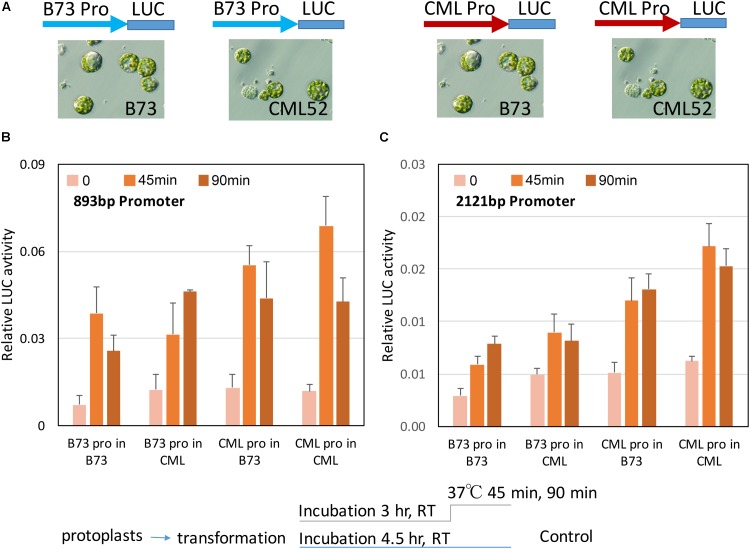
*ZmbZIP60* promoter activity in maize protoplasts. **(A)** The promoter regions from B73 (blue) or CML52 (red, same with CML247) were inserted into a pGreenII Dual-luciferase vector which has a CMV35 promoter driving Renilla luciferase as an internal control. The constructs were transformed into maize mesophyll protoplasts from B73 or CML52 as indicated. **(B,C)** 893 and 2121 bp *ZmbZIP60* promoters, respectively, were used to drive luciferase in maize protoplasts according to the scheme below. Bar graph shows means from three biological replicates. Error bars = SD.

The intrinsic activity of the different *ZmbZIP60* promoters in their response to heat stress was tested in transient assays using maize B73 and CML52 leaf protoplasts. Two different size *ZmbZIP60* promoter fragments (893 and −2121 bp upstream from the start of transcription) were amplified from CML52 (which has the same upstream sequence as the reference line, CML247) and B73. They were inserted into a pGreenII vector to drive firefly luciferase (LUC) expression. The construct also carried the Renilla luciferase gene driven by the CaMV 35S promoter that was used as an internal control. The uninduced activity of the two promoters in either B73 or CML52 protoplasts was relatively low. Following heat stress at 37°C for 45 min, the LUC levels were induced from 1.5∼4.6-fold. LUC levels were most highly induced when either the 893 or the 2121 bp CML52 promoter was used to drive expression in CML52 protoplasts. The lowest induction was when the B73 promoter was used to drive LUC expression in either B73 or CML52 protoplasts (**Figure 6**).

## Discussion

bZIP60 is a powerful transcription factor in plants driving the expression of UPR genes to alleviate ER stress (Iwata and Koizumi, 2005; Iwata et al., 2009; Nagashima et al., 2011). Heat activates plant IRE1 empowering it in maize to splice *ZmbZIP60* mRNA to make ZmbZIP60s, which, in turn, encodes the active ZmbZIP60 TF (Gao et al., 2008; Deng et al., 2011, 2016). *ZmbZIP60* is thought to autoregulate by a feed-forward mechanism, such that ER stress leads to the production of more bZIP60u RNA (Iwata and Koizumi, 2005; Humbert et al., 2012; Moreno et al., 2012). In our study, we found that the levels of ZmbZIP60s depend not only on the splicing activity of IRE1, but also on the levels of ZmbZIP60u in different maize lines. When subjected to heat stress, most of the inbred lines with *ZmbZIP60* upstream regions comparable to CML52 had higher ZmbZIP60u expression levels than those with upstream sequences comparable to B73.

We found that most tropical or subtropical maize inbred lines were more proficient than temperate lines in upregulating the expression of *ZmbZIP60* in response to heat stress. Although maize was believed to be domesticated along the tropical Pacific coast of southwest Mexico, present day growth in commercial corn is optimal at 32°C day and 27°C or below in the night (Keeling and Greaves, 1990). During the first 10–12 days after pollination, when most cell division and differentiation occurs, each 1°C increase in temperature above optimum (25°C) results in a reduction of 3–4% in grain yield (Shaw, 1983). Heat stress damages cellular components by generating reactive oxygen species and destabilizing proteins and membranes (Mittler et al., 2012; Hasanuzzaman et al., 2013; Ohama et al., 2017). Heat stress activates the cytoplasmic heat shock system, a complex transcriptional network composed of a number of transcriptional regulators and their target heat shock proteins. Although UPR is not the only heat response system in plants, nonetheless defects in the UPR demonstrate the importance of that pathway in conferring heat tolerance to various phases of plant growth (Zhang et al., 2017).

A major challenge in maize breeding is to breed for environmental stress adaptation to improve yield and seed quality. Much of today’s germplasm originated from seven progenitor lines: B73, LH82, LH123, PH207, PH595, PHG39, and Mo17 (Mikel and Dudley, 2006). The lack of genetic diversity in the inbred lines used for maize breeding limits the selection of germplasm. Since the UPR plays roles not only in the environmental stress response but also in yield and seed quality traits, understanding the natural variation in the UPR is important. In this paper, the inbred lines with higher IRE1-bZIP60 mediated UPR levels can be used to improve stiff stock lines, such as B73 or others by using marker-assistant selection or gene modification strategies. The molecular markers for the *ZmbZIP60* upstream region and ZmbZIP60u and ZmbZIP60s expression can be used to evaluate the UPR in maize germplasm and help breeders in crop improvement.

## Materials and Methods

### Plant Material and Culture Condition

Thirty-two maize inbred lines were used to correlate *ZmbZIP60* expression with the structure of the upstream region of the gene. The lines included 25 NAM founder lines and seven maize inbred lines, which have been sequenced (Mo17, F7, EP1, W22, B104 CML247, and PH207). The NAM sub-family RILs from the cross of B73 × CML52 were used in the association analysis of the factors conditioning heat induction of ZmbZIP60u. Five F1 hybrids derived from RILs (Z008E0012, Z008E0105, Z008E0127, Z008E0135, and Z008E0143) backcrossed with B73 were used to test for *cis/trans* effects. The *cis/trans* test measures the balance in expression of the two *ZmbZIP60* alleles in the hybrid in response to heat stress.

Maize seedlings were grown in soil in small pots with 13 h light/11 h dark and 26°C day/20°C night, relative humidity was set to 60%. Seedlings were tested at the three-leaf stage for variation in production and splicing of *ZmbZIP60* mRNA in response to heat stress (37°C for 1 h). For analysis of the F1 lines, 39°C for 1 h was used to induce higher levels of ZmbZIP60u for allelic analysis. Leaves were flash frozen and stored at −80°C for RNA extraction.

### RNA Extraction and RT-PCR Assay

RNA was extracted by grinding harvested tissues into powder in liquid nitrogen and using a Plant RNeasy Mini Kit (Qiagen^1^) according to the manufacturer’s instructions. 500 ng total RNA were used for the cDNA synthesis (iScript cDNA Synthesis kit (Bio-rad ^2^), which in turn was utilized as template for RT-PCR analysis. Primers used in this study were listed in **Supplementary Table S1**. Relative gene expression levels were quantified using ImageJ^3^ analysis, the expression in different lines was normalized by *ZmUbi* as an internal control. Mean values ± SD were determined from 3 or 4 biological replicates as indicated.

### *Cis/Trans* Analysis

Total RNA extraction and RT-PCR were performed as described on F1 hybrids between a cross of B73 with one of the RILs bearing the CML52 version of the *ZmbZIP60* allele. The RT-PCR products were extracted from the gel and purified using a QIAquick Gel Extraction Kit (Qiagen). After DNA sequencing, the areas under the peaks in the sequencing profile were measured using ImageJ and the ratio between the contributions by the two alleles were calculated. The average of six SNPs in this region were used to calculate the relative expression levels of allele-specific expression.

### DNA Extraction and Genotyping

Genomic DNA was isolated from maize leaves at the 3-leaf stage using a cetyltrimethylammonium bromide (CTAB) extraction method (Orkin, 1990). Primers were designed according to the sequence of reference lines (**Supplementary Table S1**).

### Transient Assay Using Maize Protoplast

Plasmids were constructed by introducing *ZmbZIP60* promoter segments into the *KpnI* and *SpeI* sites of plasmid pGreen II 8000. (SnapGene^4^). Transient expression assays were performed using maize mesophyll protoplasts as described by Sheen (2001). Luciferase activity measurements were carried out according to the kit manufacturer’s instructions (E1910, Promega, www.promega.com) and analyzed in a Berthold Centro 960 microplate luminometer.

### Sequence Analysis

DNA sequences were downloaded from maize GDB, and TEs in the *ZmbZIP60* upstream region were identified using the Maize TEs Database. Multiple sequence alignment of the reference line promoters was performed using Clustal W^5^. Primers were designed using NCBI/primer-BLAST.

### QTL Analysis

The relative expression levels of ZmbZIP60u and ZmbZIP60s were quantified with ImageJ as described in the RT-PCR assay and used as a phenotype for QTL mapping analysis performed with the R/qtl package (Broman et al., 2003). Composite interval mapping (CIM, Zeng, 1994) implemented in the package was used along with the default LOD score. 1144 legacy SNPs^6^ used in NAM population (McMullen et al., 2009) were used as markers for QTL mapping.

## Accession Numbers

*ZmbZIP60* (Zm00001d046718) and *ZmArid10* (Zm00001d046719).

## Author Contributions

ZL and SH designed the study and wrote the manuscript. ZL, RS, and JT performed all of the experiments. ZL and ZZ analyzed the data. All authors read and approved the manuscript for publication.

## Conflict of Interest Statement

The authors declare that the research was conducted in the absence of any commercial or financial relationships that could be construed as a potential conflict of interest. The reviewer SPR and handling Editor declared their shared affiliation.

## Footnotes

1 www.qiagen.com/us/

2 www.bio-rad.com

3 https://imagej.nih.gov/ij/

4 www.snapgene.com/resources/plasmid_files/plant_vectors/

5 http://www.clustal.org

6 http://www.panzea.org

## Supplementary Material

FIGURE S1 Preliminary analysis of *ZmbZIP60* (Zm00001d046718) as a biomarker for the UPR. 10 days-old maize seedlings from the temperate lines (B73 and IL14H) and subtropical lines (Ki3, CML69, CML103, and CML32) as indicated were incubated at 26°C or heat treated for 1 h at 37°C. RNA was extracted and analyzed by RT-PCR using primers specific for the unspliced form of ZmbZIP60 (ZmbZIP60u) or the spliced form (ZmbZIP60s). **(A)** RT-PCR analysis of the expression of ZmbZIP60u and ZmbZIP60s in temperate lines (B73 and IL14H) and subtropical lines (Ki3, CML69, CML103, and CML32). **(B)** The levels of expression were evaluated using ImageJ with ZmUbi as an internal control.

FIGURE S2 Ten different sequence motifs representing indels in the promoter region in near upstream region (2200 bp) of *ZmbZIP60* in the seven different maize inbred lines. Sequence form six public lines and the inbred line used in this study were used for multiple sequence alignment.

FIGURE S3 One-way ANOVA analysis, multiple comparisons of the data in **Figures 5C,D** and correlation analysis of the CACTA-TE/Motif 5, Motif 4, and the interaction of all to the *ZmbZIP60* induction levels using a linear model.

TABLE S1 Primers used in this study.
