# Supplementary figures and images for: Cis-Effects Condition the Induction of a Major Unfolded Protein Response Factor, ZmbZIP60, in Response to Heat Stress in Maize
Source: Front Plant Sci. 2018 Jun 29;9:833. doi: 10.3389/fpls.2018.00833 (PMC6034121; doi:10.3389/fpls.2018.00833)

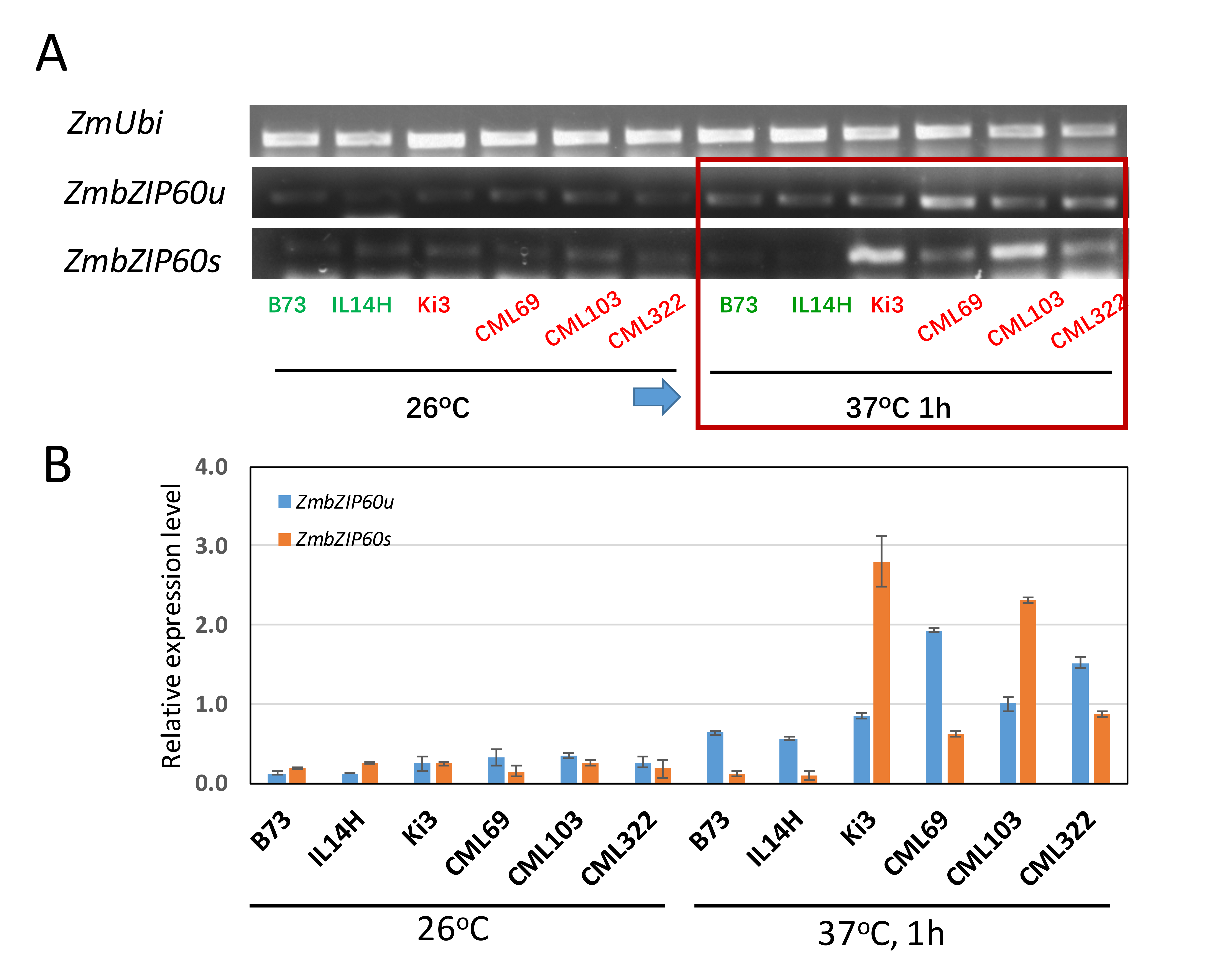

Supplement: FIGURE S1 — Preliminary analysis of ZmbZIP60 (Zm00001d046718) as a biomarker for the UPR. 10 days-old maize seedlings from the temperate lines (B73 and IL14H) and subtropical lines (Ki3, CML69, CML103, and CML32) as indicated were incubated at 26°C or heat treated for 1 h at 37°C. RNA was extracted and analyzed by RT-PCR using primers specific for the unspliced form of ZmbZIP60 (ZmbZIP60u) or the spliced form (ZmbZIP60s). (A) RT-PCR analysis of the expression of ZmbZIP60u and ZmbZIP60s in temperate lines (B73 and IL14H) and subtropical lines (Ki3, CML69, CML103, and CML32). (B) The levels of expression were evaluated using ImageJ with ZmUbi as an internal control. [file Image_1.TIF]
