# Supplementary material for: Cis-Effects Condition the Induction of a Major Unfolded Protein Response Factor, ZmbZIP60, in Response to Heat Stress in Maize
Source: Front Plant Sci. 2018 Jun 29;9:833. doi: 10.3389/fpls.2018.00833 (PMC6034121; doi:10.3389/fpls.2018.00833)
Supplement: FIGURE S2 — Ten different sequence motifs representing indels in the promoter region in near upstream region (2200 bp) of ZmbZIP60 in the seven different maize inbred lines. Sequence form six public lines and the inbred line used in this study were used for multiple sequence alignment. [file Image_2.pdf]

```
Motif 1
EP1       accgcccactaaactgccaggaacgaggaagcttccttcctctccttcagtccttcctcg   -80
F7        accgcccactaaactgccaggaacgaggaagcttccttcctctccttcagtccttcctcg   -80
CML52     accgcccactaaactgccaggaacgaggaagcttccttcctctccttcagtccttcctcg   -80
CML247    accgcccactaaactgccaggaacgaggaagcttccttcctctccttcagtccttcctcg   -80
PH207     accgcccactaaactgccaggaacgaggaagcttccttcctctccttcagtccttcctcg   -80
B73       accacccactaaactgccactctgccaggaacgaggaagcttccttcctgtccttcctcg   -80
B104      accacccactaaactgccactctgccaggaacgaggaagcttccttcctgtccttcctcg   -80
          *** ***************          * * *         **   * * * ***********

Motif 2
EP1       ccgcgcacagcgacaggcagcattccttgccgcagtgagctcaagatcccgggtgatctg   -175
F7        ccgcgcacagcgacaggcagcattccttgccgcagtgagctcaagatcccgggtgatctg   -175
CML52     ccgcgcacagcgacaggcagcattccttgccgcagtgagctcaagatcccgggtgatctg   -175
CML247    ccgcgcacagcgacaggcagcattccttgccgcagtgagctcaagatcccgggtgatctg   -175
PH207     ccgcgcacagcgacaggcagcattccttgccgcagtgagctcaagatcccgggtgatctg   -175
B73       ccgcgcacaagaacccctgcgacgggca----gcaaagcattccttgccggagtgagctg   -175
B104      ccgcgcacaagaacccctgcgacgggca----gcaaagcattccttgccggagtgagctg   -175
          *********   **       *                  *      ** * **** ***

Motif 3
EP1       gcacgggagtatcaaatacgtgccatcgcccatcgccggtgcttagaaactgacggtcgg   -270
F7        gcacgggagtatcaaatacgtgccatcgcccatcgccggtgcttagaaactgacggtcgg   -270
CML52     gcacgggagtatcaaatacgtgccatcgcccatcgccggtgcttagaaactgacggtcgg   -270
CML247    gcacgggagtatcaaatacgtgccatcgcccatcgccggtgcttagaaactgacggtcgg   -270
PH207     gcacgggagtatcaaatacgtgccatcgcccatcgccggtgcttagaaactgacggtcgg   -270
B73       gcacgggagtatgaaatacgtgccatc-------gccggtgcttcgaaactgacggtcgg   -266
B104      gcacgggagtatgaaatacgtgccatc-------gccggtgcttcgaaactgacggtcgg   -266
          ************ **************       ********** ***************

Motif 4
EP1       cttggtcccatatgaattatg------------gttatgcgtgtaggtcgtgctgtgtag   -477
F7        cttggtcccatatgaattatg------------gttatgcgtgtaggtcgtgctgtgtag   -477
CML52     cttggtcccatatgaattatg------------gttatgcgtgtaggtcgtgctgtgtag   -477
CML247    cttggtcccatatgaattatg------------gttatgcgtgtaggtcgtgctgtgtag   -477
PH207     cttggtcccatatgaattatg------------gttatgcgtgtaggtcgtgctgtgtag   -477
B73       cttgatctcctatgaatttaggtactgtgtccgccacttgttgcagatcgtgctgtatag   -466
B104      cttgatctcctatgaatttaggtactgtgtccgccacttgttgcagatcgtgctgtatag   -466
          ****  ** * ********   *              *    ** ** ********* ***

Motif 5
EP1       tattgatataaaacttgcattgtatgcaacagtagtttttcgatattacttaatttggtc   -535
F7        tattgatataaaacttgcattgtatgcaacagtagtttttcgatattacttaatttggtc   -535
CML52     tattgatataaaacttgcattgtatgcaacagtagtttttcgatattacttaatttggtc   -535
CML247    tattgatataaaacttgcattgtatgcaacagtagtttttcgatattacttaatttggtc   -535
PH207     tattgatataaaacttgcattgtatgcaacagtagtttttcgatattacttaatttggtc   -535
B73       tattaatataa-----------------------aacttggattgttacttagtttggtc   -536
B104      tattaatataa-----------------------aacttggattgttacttagtttggtc   -536
          **** ******                       **     * ******** *******

Motif 6
EP1       gttgttcaaggtgatagacaacttctagttagaacttag----------------cggta   -638
F7        gttgttcaaggtgatagacaacttctagttagaacttag----------------cggta   -638
CML52     gttgttcaaggtgataaacaacttctagttagaacttag----------------cggta   -638
CML247    gttgttcaaggtgataaacaacttctagttagaacttag----------------cggta   -638
PH207     gttgttcaaggtgataaacaacttctagttagaacttag----------------cggta   -638
B73       accgttcaaggagatagacattcgataggtacgtacaacttctagctagaatttagggac   -623
B104      accgttcaaggagatagacattcgataggtacgtacaacttctagctagaatttagggac   -623
             ******** **** ***      *** **       *                **

EP1       atgtttgtttcttttgtttatattttttttaact-------cgtatcatatattgatata   -585
F7        atgtttgtttcttttgtttatattttttttaact-------cgtatcatatattgatata   -585
CML52     atgtttgtttcttttgtttatatattttttaact-------cgtatcatatattgatata   -585
CML247    atgtttgtttcttttgtttatatattttttaact-------cgtatcatatattgatata   -585
PH207     atgtttgtttcttttgtttatatattttttaact-------cgtatcatatattgatata   -585
B73       atgtttgtttcttttgtttatatagtttttttcttttaacttgtatcgagtattaatata   -563
B104      atgtttgtttcttttgtttatatagtttttttcttttaacttgtatcgagtattaatata   -563
          ***********************   *****   **         *****  **** *****

Motif 7
EP1       ctgaatgtgaggcttgaccgtt----------------gcataggggaggcaaaggtggc   -1134
F7        ctgaatgtgaggcttgaccgtt----------------gcataggggaggcaaaggtggc   -1134
CML52     ctgaatgtgaggcttgaccgtt----------------gcataggggaggcaaaggtggc   -1133
CML247    ctgaatgtgaggcttgaccgtt----------------gcataggggaggcaaaggtggc   -1133
PH207     ctgaatgtgaggcttgaccgtt----------------gcataggggaggcaaaggtggc   -1133
B73       ctgaatgtgaggcttgactgttgcacgcagtggcggatccataggggaggcaaaggtggc   -1133
B104      ctgaatgtgaggcttgactgttgcacgcagtggcggatccataggggaggcaaaggtggc   -1133
          ****************** ***                 *********************

Motif 8
EP1       tggttgtttcaatcgacgatac--cttcatatgaattatagggagtctgtatgatttgta   -1446
F7        tggttgtttcaatcgacgatac--cttcatatgaattatagggagtctgtatgatttgta   -1446
CML52     tggttgtttcaatcgacgatac--cttcatatgaattatagggagtctgtatgatttgta   -1445
CML247    tggttgtttcaatcgacgatac--cttcatatgaattatagggagtctgtatgatttgta   -1445
PH207     tggttgtttcaatcgacgatac--cttcatatgaattatagggagtctgtatgatttgta   -1445
B73       tggttgtttcaattgatggtaccccttcatacaaattacagggattctggatgatttgta   -1460
B104      tggttgtttcaattgatggtaccccttcatacaaattacagggattctggatgatttgta   -1460
          *************  ** * ***   *******   ***** ***** **** **********

Motif 9
EP1       catctaggaggagggatcttggggttgccttgggggtcggtaagccatccaaagcgccgc   -1624
F7        catctaggaggagggatcttggggttgccttgggggtcggtaagccatccaaagcgccgc   -1624
CML52     catctaggaggagggatcttggggttgccttggggatcggtaagtcatccaaagcgccgc   -1623
CML247    catctaggaggagggatcttggggttgccttggggatcggtaagtcatccaaagcgccgc   -1623
PH207     catctaggaggagggatcttggggttgccttggggatcggtaagtcatccaaagcgccgc   -1623
B73       catctaggaggg---atc-ttggggttgccttggggtcggtaagccatccaaagcgctgc   -1640
B104      catctaggaggg---atc-ttggggttgccttggggtcggtaagccatccaaagcgctgc   -1640
          ***********    *** * *** *   * * *** ******** ************ **

Motif 10
EP1       gataggtcacttggcaca-------------tagagctcctggaagaacccagatcctgc   -1700
F7        gataggtcacttggcaca-------------tagagctcctggaagaacccagatcctgc   -1700
CML52     gataggtcacttggcaca-------------tagagctcctggaagaacccagatcctgc   -1699
CML247    gataggtcacttggcaca-------------tagagctcctggaagaacccagatcctgc   -1699
PH207     gataggtcacttggcaca-------------tagagctcctggaagaacccagatcctgc   -1699
B73       gataggtcactagaccgtccgcgatggcacatagagctctagaaagaacctagaccccgc   -1712
B104      gataggtcactagaccgtccgcgatggcacatagagctctagaaagaacctagaccccgc   -1712
          *********** * *                 ********   * ******* *** ** **
```

Figure S2
