# Supplementary material for: Cis-Effects Condition the Induction of a Major Unfolded Protein Response Factor, ZmbZIP60, in Response to Heat Stress in Maize
Source: Front Plant Sci. 2018 Jun 29;9:833. doi: 10.3389/fpls.2018.00833 (PMC6034121; doi:10.3389/fpls.2018.00833)
Supplement: FIGURE S3 — One-way ANOVA analysis, multiple comparisons of the data in Figures 5C,D and correlation analysis of the CACTA-TE/Motif 5, Motif 4, and the interaction of all to the ZmbZIP60 induction levels using a linear model. [file Image_3.pdf]

One-way ANOVA analysis and multiple comparisons by using Tukey test of Figure 5C and 5D

Figure 5C

	Df	Sum Sq	Mean Sq	F value	Pr(>F)
Groups	2	3.17E+08	158555825	12.68	1.35e-05 ***
Residuals	93	1.16E+09	12502743		

Signif. codes: 0 ‘***’ 0.001 ‘**’ 0.01 ‘*’ 0.05 ‘.’ 0.1 ‘ ’ 1

comparison	P value adjust
CML type-B73 type	0.0002496
other-B73 type	0.7136741
CML-Other	0.0006506

Figure 5D

	Df	Sum Sq	Mean Sq	F value	Pr(>F)
Groups	3	4.39E+08	146429067	12.95	3.97e-07 ***
Residuals	92	1.04E+09	11310648		

Signif. codes: 0 ‘***’ 0.001 ‘**’ 0.01 ‘*’ 0.05 ‘.’ 0.1 ‘ ’ 1

comparison	P value adjust
CML type-B73 type	0.0000017
Other-B73 type	0.0092124
B73 ike-B73 type	0.9681291
Other-CML type	0.904658
B73-like-CML type	0.0024962
B73-like-Other	0.0404958

Correlation analysis of the CACTA-TE/Motif 5, Motif 4 and the interaction of all to the ZmbZIP60 expression levels by using linear model

ZmbZIP60 expression levels~(CACTA-TE/motif 5+motif 4+CACTA-TE/motif 5*motif 4)

Coefficients:

	Estimate	Std. Error	t value	Pr(>|t|)	
(Intercept)	-6021.7	3122.2	-1.929	0.05685	
CACTA-TE/motif 5	3230.1	1227.9	2.631	0.00999	**
motif 4	11314.2	2234.1	5.064	2.10E-06	***
Interaction	-3510.8	749.1	-4.687	9.58E-06	***

Signif. codes: 0 ‘***’ 0.001 ‘**’ 0.01 ‘*’ 0.05 ‘.’ 0.1 ‘ ’ 1

Figure S3
