# Supplementary material for: Cis-Effects Condition the Induction of a Major Unfolded Protein Response Factor, ZmbZIP60, in Response to Heat Stress in Maize
Source: Front Plant Sci. 2018 Jun 29;9:833. doi: 10.3389/fpls.2018.00833 (PMC6034121; doi:10.3389/fpls.2018.00833)
Supplement: TABLE S1 — Primers used in this study. [file Table_1.PDF]

Table S1 Primers used in this paper

	Gene name	V3_ID_maize	V4_ID_maize	Forward primer (5' to 3')	Reverse primer (5' to 3')
Primers used ZmbZIP60 promoter analysis	Motif 4	B73 Motif 4specific		CAAGGTCGATGTCGGGGAAG	ATTTAGGTACTGTGTCCGCCAC
		CML Motif 4specific		GTCGATGTCCATGGGAACGG	CCCATATGAATTATGGTTATGCG
	Motif 5	B73 Motif 5specific		CAAGGTCGATGTCGGGGAAG	ATATAAAACTTGGATTGTTACTTAG
		CML Motif 5specific		GTCGATGTCCATGGGAACGG	ACTTGCATTGTATGCAACAGTAGT
	CACAT TE	B73 CACAT TE specific		GGCCATGCGTTGAGCATTAG	ATGTCAGAGCTTGAGACTTATGTT
		CML promoter specific		CAAAGGTCGCCCTAACGTCT	GACGAGACGAATGTAGCGGT
Primers used fro RT-PCR analysis	ZmbZIP60	GRMZM2G025812	Zm00001d046718	GCAGAGTGCCGTCGCCTCAGCTAC	CCAGCCAAAGCAGGGAAACCAGC
		Unspliced form		GCAGAGTGCCGTCGCCTCAGCTAC	GGCAGGGTTTCCGTGAGTAC
		Spliced form		GCAGAGTGCCGTCGCCTCAGCTAC	GCAGGGAAACCAGCGGCTGAC
		Allele specific analysis		ACGGCACCACCCACTAAACT	CCTGCATCAGGAGCCTCTCG
	Ubiquitin	GRMZM2G409726	Zm00001d010159	TAAGCTGCCGATGTGCCTGCGTCG	CTGAAAGACAGAACATAATGAGCACAG
	Actin1	GRMZM2G126010	Zm00001d015327	ATCACCATTGGGTCAGAAAGG	GTGCTGAGAGAAGCCAAAATAGAG
